# A model based on electronic health records to predict transfusion events in on-pump cardiac surgery

**DOI:** 10.1016/j.isci.2023.107798

**Published:** 2023-09-01

**Authors:** Dong Xu Chen, Yi Shun Wang, Min Yan, Lei Du, Qian Li

**Affiliations:** 1 Department of Anesthesiology, West China Hospital, Sichuan University, No. 37 Wainan Guoxue Road, Chengdu, Sichuan 610041, P.R.China; 2 The Research Units of West China (2018RU012)-Chinese Academy of Medical Sciences, West China Hospital, Sichuan University, No. 37 Wainan Guoxue Road, Chengdu, Sichuan 610041, P.R.China; 3 Department of Anesthesiology, The Second Affiliated Hospital, Zhejiang University, Hangzhou, Zhejiang 330100, P.R.China

**Keywords:** Cardiovascular medicine, Surgical procedure, Bioinformatics

## Abstract

Perioperative blood transfusion is costly and raises safety concerns. We developed and validated a model for predicting minor, moderate, or major transfusion given to patients during on-pump cardiac procedures based on two centers’ database. Model performance incorporating 7 variables on the development set had an AUC of 0.803 [95% CI, 0.790–0.815] for minor transfusion; moderate transfusion, giving an AUC of 0.822 (95% CI, 0.803–0.841); and major transfusion, giving an AUC of 0.813 (95% CI, 0.759–0.866). Model performance on the validation set had an AUC of 0.739 (95% CI 0.714–0.765), 0.730 (95% CI 0.702–0.758), and 0.713 (95% CI 0.677–0.749), respectively. A model based entirely on readily available electronic health records can accurately predict intraoperative minor, moderate, or major transfusion and provide individualized transfusion risk profiles before surgery among those on-pump cardiac surgical patients, and may help guide patient management.

## Highlights

• We proposed a model to predict minor, moderate, or major transfusion
• We measured generalizability of the model in an external cohort
• Our model showed good performance in the development and validation cohorts
• Our model may help personalize cardiac surgery to improve patient safety

## Graphical abstract

**Figure:**
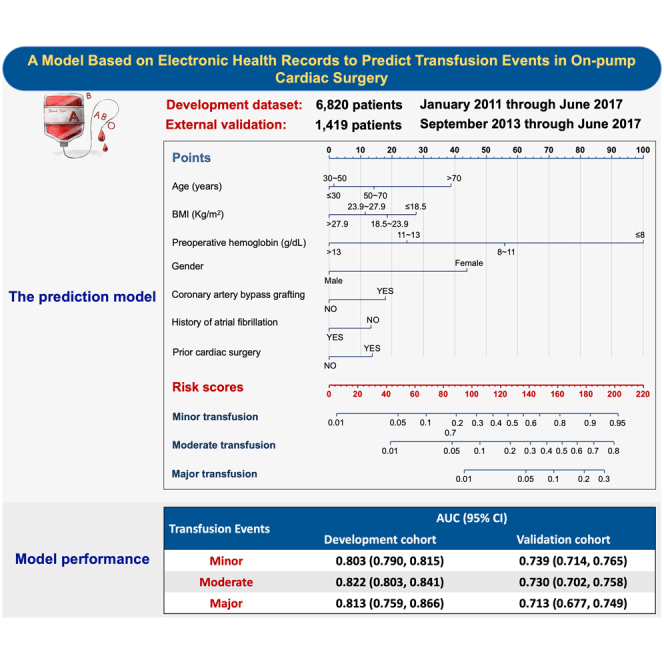

## Introduction

Approximately 2 million patients undergo cardiac surgeries annually worldwide,^1^ and up to 50% of those patients receive perioperative red blood cell (RBC) transfusion,^2^^,^^3^ making them the “most transfused” group of surgical patients.^4^^,^^5^ Transfusion increases risk of morbidity and mortality after cardiac surgery in proportion to the RBC volume transfused,^2^^,^^6^ so it should be performed only when necessary.^7^ In addition, it consumes valuable medical resources, so being able to predict which patients will require transfusion, and how many units they will need, may help manage rationalize the use of blood supplies.^7^ Optimal transfusion strategies can decrease postoperative morbidity and shorten the hospital length of stay, especially in patients at high risk of transfusion.^8^^,^^9^^,^^10^ Rationalizing transfusions may also make clinical trials more rigorous, since heterogeneous requirements for transfusion can confound analyses in surgical studies.^11^^,^^12^

Several models have been described that can predict whether or not a given patient will require perioperative RBC transfusion. These models have limitations because they cannot predict the transfusion units;^13^^,^^14^^,^^15^^,^^16^ they have not been externally validated (such as MBT prediction model, and TRUST)^14^^,^^15^^,^^16^ or they have not performed well in external cohorts (such as the TRACK score).^13^ As a result, these models are not widely used in the clinic.

Here, we created a model to develop a more comprehensive model that could predict the different volumes of intraoperative RBC transfusion in on-pump cardiac surgery patients, based on straightforward extraction of data in electronic health records. We also examined the relationship between transfusion units during surgery and postoperative adverse events. We validated the model in an external cohort from a different medical center. We hypothesize that our prediction model incorporating electronic health record would yield satisfactory performance in predicting the different volume of intraoperative RBC transfusion in on-pump cardiac surgery patients.

## Results

### Cohort characteristics

Of the 6,820 patients from the development cohort, and 1,419 in the external validation cohort (Figure S1). The mean age in the development cohort was 51.2 years (SD 10.9) and 2744 (40.2%) of study patients were male (Table 1; Table S1). Besides, external validation cohort had a mean age of 57.8 years (SD 11.3) and 52.4% (744) were men. Most patients underwent valvular related surgical, and the mean operative time was more than 4 h in both cohorts. The two cohorts varied substantially in most of the measured variables, specially, patients in validation cohort generally showing worse health.

**Table 1:** Baseline characteristics of patients in the development and external validation cohorts

Characteristic	Development cohort N = 6820	External validation cohort N = 1419	p value
**Baseline characteristics**			

Age (years), mean (SD)	51.19 (10.90)	57.78 (11.32)	<0.001
Sex, n (%)			<0.001
Male	2744 (40.2)	744 (52.4)	
Female	4076 (59.8)	675 (47.6)	
Ethnicity, n (%)			<0.001
Han Chinese	6625 (97.1)	1415 (99.7)	
Tibetan	101 (1.5)	2 (0.1)	
Other	94 (1.4)	2 (0.1)	
BMI (kg.m^2^-1), mean (SD)	22.94 (3.18)	22.61 (3.27)	<0.001
Current alcohol, n (%)	1282 (18.8)	245 (17.3)	0.189
Current smoker, n (%)	4955 (72.7)	982 (69.2)	0.009

**Comorbidities and medical history, n (%)**			

Asthma	18 (0.3)	5 (0.4)	0.578
COPD	44 (0.6)	23 (1.6)	0.001
Pneumonia	64 (0.9)	68 (4.8)	<0.001
Atelectasis	21 (0.3)	4 (0.3)	1
Hydrothorax	101 (1.5)	37 (2.6)	0.004
Cerebral infarction	235 (3.4)	103 (7.3)	<0.001
Cerebral hemorrhage	16 (0.2)	5 (0.4)	0.39
Diabetes	384 (5.6)	146 (10.3)	<0.001
Hyperthyroidism	109 (1.6)	23 (1.6)	0.908
Hypothyroidism	1249 (18.3)	46 (3.2)	<0.001
Liver insufficiency	399 (5.9)	24 (1.7)	<0.001
Renal dysfunction	1 (0.0)	18 (1.3)	<0.001
Gastrointestinal bleeding	26 (0.4)	6 (0.4)	0.814
Hypertension	865 (12.7)	479 (33.8)	<0.001
Hyperlipemia	1761 (25.8)	451 (31.8)	<0.001
CAD			<0.001
No or Unknown	6179 (90.6)	1112 (78.4)	
Single vessel involved	165 (2.4)	67 (4.7)	
Double vessels involved	143 (2.1)	51 (3.6)	
Triple vessels involved	333 (4.9)	189 (13.3)	
Prior endocarditis	146 (2.1)	47 (3.3)	0.012
Pulmonary hypertension			<0.001
Mild	273 (4.0)	209 (14.7)	
Moderate	594 (8.7)	164 (11.6)	
Severe	289 (4.2)	53 (3.7)	
Atrial fibrillation	3246 (47.6)	504 (35.5)	<0.001
Peripheral vascular disease	72 (1.1)	40 (2.8)	<0.001
Congestive heart failure	1470 (21.6)	53 (3.7)	<0.001
Prior cardiovascular surgery	1435 (21.0)	398 (28.0)	<0.001

**NYHA classification, n (%)**			

I	67 (1.0)	82 (5.8)	<0.001
II	1528 (22.4)	580 (40.9)	
III	5072 (74.4)	633 (44.6)	
IV	153 (2.2)	124 (8.7)	

**ASA physical status, n (%)**			

II	114 (1.7)	192 (13.5)	<0.001
III	6312 (92.6)	1120 (78.9)	
IV	394 (5.8)	107 (7.5)	

**Surgery type, n (%)**			

Single valve replacement	2729 (40.0)	618 (43.6)	0.015
Multiple valve replacement	3468 (50.9)	474 (33.4)	<0.001
CABG	553 (8.1)	246 (17.3)	<0.001
Combined CABG and value replacement	70 (1.0)	81 (5.7)	<0.001
Radiofrequency ablation	1615 (23.7)	163 (11.5)	<0.001
Atrial thrombus removal	796 (11.7)	74 (5.2)	<0.001

**Intraoperative data**			

Operation time (h), mean (SD)	4.95 (1.12)	4.39 (1.59)	<0.001
CPB time (min), mean (SD)	117.95 (38.61)	128.05 (55.74)	<0.001
Aortic cross-clamping time (min), mean (SD)	79.00 (30.83)	90.83 (42.57)	<0.001
Total transfusion of RBC (U), median [IQR]	0.00 [0.00, 3.00]	2.00 [0.00, 4.00]	<0.001
Intraoperative transfusion of RBC volume (U), median [IQR]	0.00 [0.00, 0.00]	2.00 [0.00, 4.00]	<0.001
Postoperative transfusion of RBC volume (U), median [IQR]	0.00 [0.00, 2.00]	0.00 [0.00, 0.00]	<0.001
Transfusion of thrombin (U), median [IQR]	0.00 [0.00, 2.00]	0.00 [0.00, 0.00]	<0.001
Residual pump blood after CPB (mL), median [IQR]	600.00 [600.00, 800.00]	500.00 [500.00, 680.00]	<0.001

Continuous variables were reported as mean (standard deviation) or median (interquartile range); categorical variables, as n (%).

ASA, American Society of Anesthesiologists; BMI, body mass index; CAD, coronary artery disease; CABG, coronary artery bypass grafting; COPD, chronic obstructive pulmonary disease; CPB, cardiopulmonary bypass; IQR, interquartile range; NYHA, New York Heart Association; RBC, red blood cell; SD, standard deviation.

In the development cohort, 1219 (17.9%) patients received RBC transfusion, and 69 (1.0%) of all patients received major RBC transfusion (Table S2). In the external validation cohort, 749 (52.8%) patients received RBC transfusion, and 230 (16.2%) of all patients received major RBC transfusion. Variables with missing data more than 10% of patients are presented in Figure S2.

### Selection of predictors for the model

Uni- and multivariable ordinal logistic regression was conducted to identify candidate predictors associated with the different volumes of RBC transfusion (Table S3). Multivariable analysis identified 7 candidate predictors (Figure S4A).

**Figure 1:**
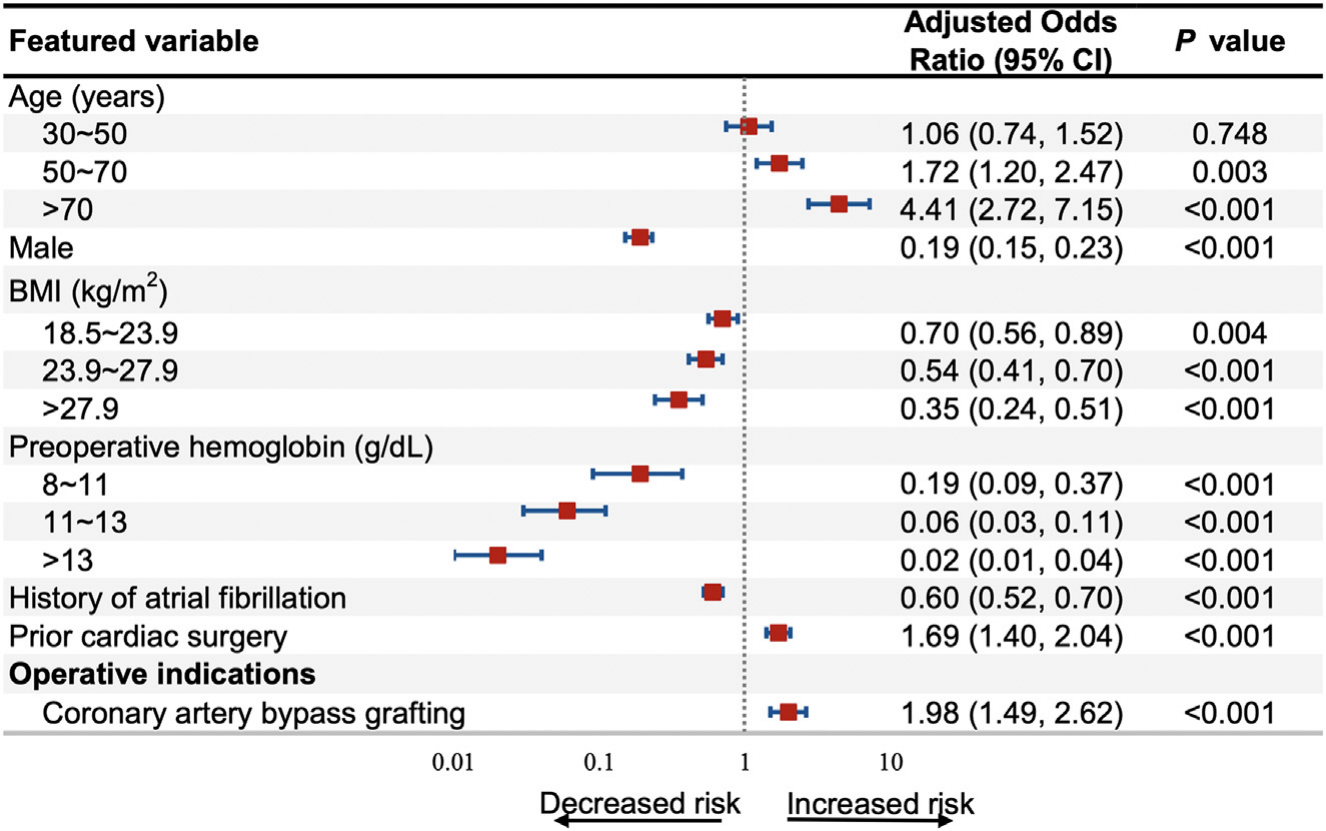
All candidate predictors included in the final model Age, BMI, and preoperative hemoglobin were non-linear and were split into 3 categorical variables based on restricted cubic splines with 3–5 knots.

Of all variables originally included in the LASSO algorithm (Figure S3A), only 14 candidate predictors were retained as λ increased to 0.018, which was one standard error of the minimum λ (Figures S3B). Therefore, 14 candidate predictors were identified by LASSO algorithm (Figure S4B). Comparisons of the ordinal logistic regression and LASSO algorithms in terms of AUCs and other metrics showed that the two were similarly accurate at predicting different transfusion volume (Table 2), although LASSO predicted minor and moderate transfusion significantly better (p < 0.001). The multivariable ordinal logistic regression model with 7 predictors showed the well performance (Figure 1).

**Table 2:** Comparison of two approaches for selecting predictors of transfusion in the development cohort

	Uni- and multivariable stepwise regression	Least absolute shrinkage and selection	*P*
**Number of predictors**	7	14	
**Area under the receiver operating characteristic curve (95% confidence interval)**			
Minor transfusion	0.816 (0.804 0.828)	0.825 (0.814 0.837)	<0.001
Moderate transfusion	0.836 (0.817 0.854)	0.843 (0.825 0.861)	0.010
Major transfusion	0.839 (0.787 0.891)	0.831 (0.780 0.883)	0.176
**Overall accuracy (95% confidence interval)**	0.822 (0.813, 0.831)	0.822 (0.813, 0.831)	–
**Model performance**			
Nagelkerke R^2^	0.272	0.286	–
Root mean squared error	0.685	0.686	–
Bayesian information criterion	6970.62	6965.85	–

**Figure 2:**
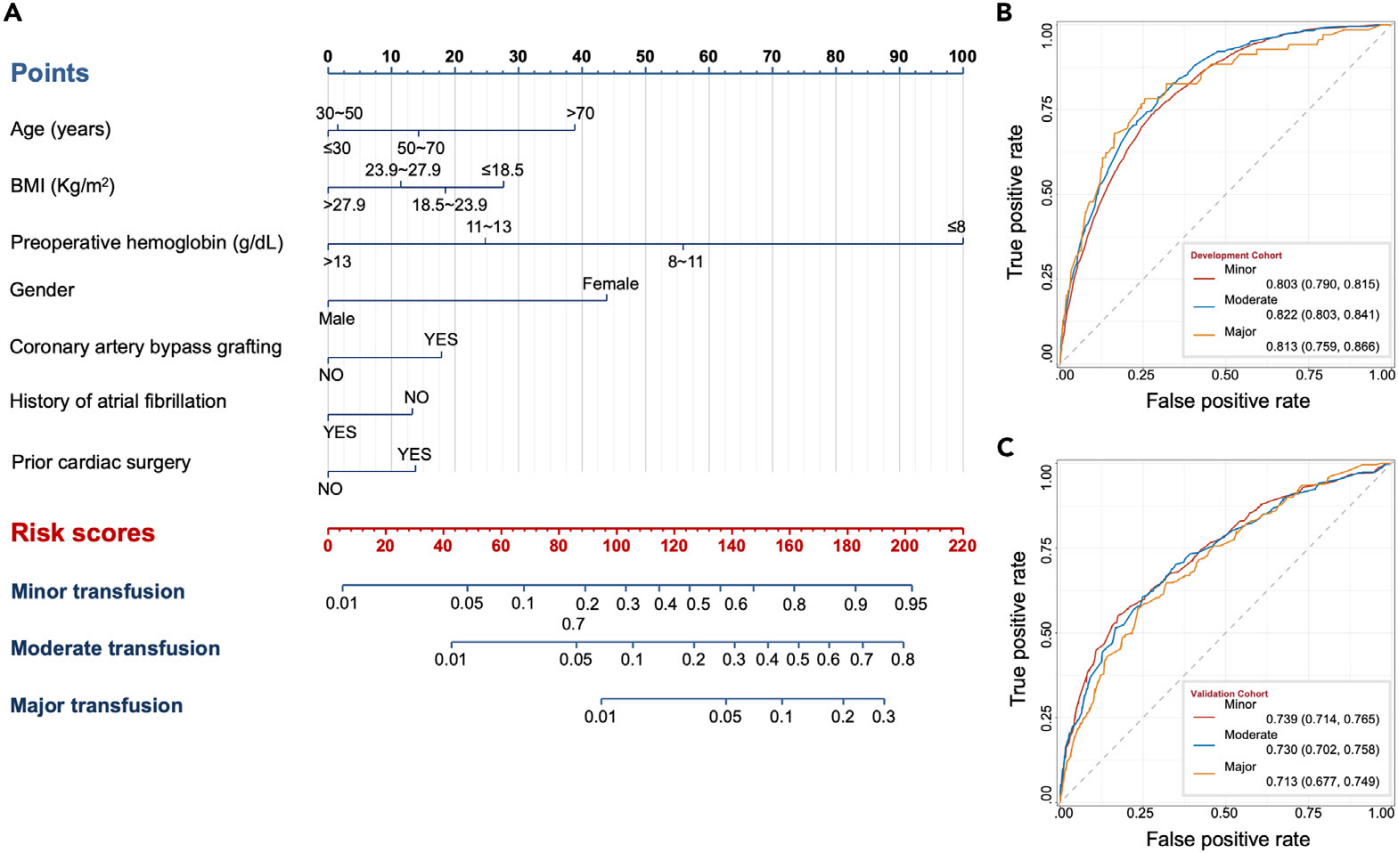
The prediction model (A) Depiction of the model. (B and C) Receiving operating curves of our model in the (B) development cohort or (C) external validation cohort.

For the final prediction model, the nonlinear continuous predictors of age, body mass index (BMI) and hemoglobin were split into categorical predictors using RCS models (Figure S5). Total 7 predictors were integrated into transfusion risk estimation nomogram (Figure 2A). The predictors were as follows: age (≤30, 30–50, 50–70, >70 years), sex (male vs. female), BMI (≤18.5, 18.5–23.9, 23.9–27.9, >27.9 kg/m^2^), preoperative hemoglobin (≤8, 8–11, 11–13, >13 g/dL), atrial fibrillation, history of cardiac surgery, and surgery indication (i.e., CABG). The ORs and 95% CIs for the predictors are shown in Figure 2. No predictor violated the VIF criteria in the final ordinal logistic model. Before the prediction modeling, the parallel assumption in ordinal logistic regression was tested by a Brant-Wald test. Results from the Brant-Wald test were supplemented in Table S4, which demonstrated that all predictors in the final model met the assumptions with p > 0.05.

Current predictive models can be used following the steps: First, determining the patient’s point for each predictive variable. Second, drawing a straight line upwards from each predictive point to the top point reference line. Third, summing the points from each predictor. Fourth, locating the sum on the total points reference line, and drawing a straight line from total points line down to the bottom probability line to find the patient’s likelihood of transfusion.

In addition to using Nomogram directly to characterize risk of transfusion, we also provide risk calculation equation as shown below:

Minor transfusion = −3.08e-07 ∗ points ˆ3 + 0.000112184 ∗ points ˆ2 + −0.005586625 ∗ points + 0.049579306.

Moderate transfusion = −1.34e-07 ∗ points ˆ3 + 8.642e-05 ∗ points ˆ2 + −0.009116193 ∗ points + 0.25776949.

Major transfusion = 3.04e-07 ∗ points ˆ3 + −9.2098e-05 ∗ points ˆ2 + 0.009878439 ∗ points + −0.357488222.

### Model development, validation, and explanation

The final model with an AUC of 0.803 (95% CI 0.790–0.815) for minor transfusion, 0.822 (95% CI 0.803–0.841) for moderate transfusion, and 0.813 (95% CI 0.759–0.866) for major transfusion (Figure 2B). Good agreement was observed through calibration curve between predicted and observed outcomes. Specially, for minor transfusion, the Brier score was 0.120 and the calibration slope was 0.995 (Figure S6A); moderate transfusion, 0.050 and 1.010 (Figure S6B); and major transfusion, 0.010 and 0.916 (Figure S6C).

During the external validation process, the model predicted had an AUC of 0.739 (95% CI 0.714–0.765) for minor transfusion; moderate transfusion, 0.730 (95% CI 0.702–0.758); and major transfusion, 0.713 (95% CI 0.677–0.749) (Figure 2C). Besides, the respective Brier scores were 0.294, 0.261, and 0.150 and respective calibration slopes were 0.662, 0.611, and 0.517 (Figures S6D–S6F). Notably, calibration of this model was limited in the validation cohorts. The Brier scores demonstrate that forecast is less accurate in minor or moderate transfusion in externally validated process, and optimization of the model is needed.

**Figure 3:**
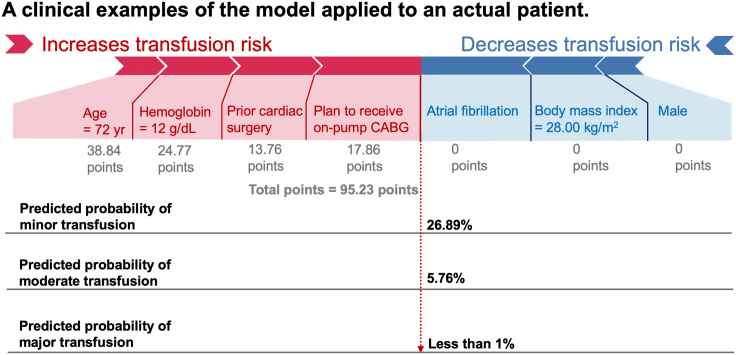
Example of application of the prediction model to an individual patient A man (0 points), aged 72 years (38.84 points), and undergoes on-pump CABG (17.86 points). He had a preoperative hemoglobin level of 12 g/dL (24.77 points) and a history of prior cardiac surgery (13.76 points). His total risk score is 95.23 points, corresponding to a 26.89% probability of receiving minor transfusion, 5.76% probability of receiving moderate transfusion, and less than 1% probability of receiving major transfusion.

Figure 3 illustrates how the model yields predictions for an individual patient. Decision curve analysis indicated that, the prediction model would provide more clinical benefit than either the “treat all patients” scenario or the “treat no patient” scenario when the probability of any transfusion volume (minor, moderate, major) was greater than 2% (Figures S7A–S7C) in the development cohort. In the external validation cohort, the corresponding threshold probabilities were 45%, 32%, 16% for minor, moderate, and major transfusion, respectively (Figures S7D–S7F).

As an additional measure of the reliability of our results, we used an on–line tool (https://riskcalc.org/samplesize/)^17^ to estimate, *post hoc*, the minimal sample needed to predict the three categories of transfusion with the final model. Based on a Nagelkerke R^2^ of 0.272 and RBC transfusion rate of 17.9% in the development cohort, we would need a cohort of at least 483 patients and a minimum of 8.8 events per predictor. Thus, our development cohort of 6,820 patients should be large enough to provide reliable results.

### The association of transfusion volume and composite adverse events

The incidence of transfusion and postoperative composite adverse events in each cohort are shown in Table S2. In the development cohort, we identified 1003 (14.7%) patients with postoperative composite adverse events, significantly higher than the 630 (44.4%) in the external validation cohort (p < 0.001).

Before IPTW, the risk of composite adverse events relative to the risk without any transfusion was OR 1.34 (95% CI 1.11–1.63) among patients who received minor transfusion, 1.95 (95% CI 1.49–2.52) among those who received moderate transfusion, and 2.78 (95% CI 1.62–4.61) among those who received major transfusion (Figure S7). Risk increased significantly as transfusion volume increased (*P* for trend <0.001). This tendency remained significant (*P* for trend <0.001; Figure S9) after IPTW balanced the relative influences of baseline variables (Figures S8A–S8B). The adjusted IPTW–weighted ORs were 1.33 (95% CI 1.19–1.48) for minor transfusion, 1.70 (95% CI 1.46–1.98) for moderate transfusion, and 1.92 (95% CI 1.42–2.63) for major transfusion.

## Discussion

The present study improves on the literature (e.g., our model can predict the minor, moderate, major transfusion and validate in externally medical center)^13^^,^^15^^,^^18^ by describing an accurate model for predicting intraoperative different RBC transfusion volume during on-pump cardiac surgery, based entirely on data easily accessible from electronic medical records. The model was developed with data from 6820 patients at a major medical center in China, then validated in an independent cohort of 1419 patients at another center in the country. This model may help clinicians stratify patients by transfusion risk and rationalize treatment decisions. In addition, our study confirmed that risk of adverse events increases with RBC transfusion volume.

Unlike previous models for predicting transfusion, our model can predict transfusion volume, at least by category (minor, moderate, or major). Prognosis can differ substantially between patients receiving small or large transfusion volumes.^2^^,^^6^^,^^14^ For example, incidence of infections and ischemic events may be several–fold higher among those receiving >4 RBC units than among those receiving ≤2 units.^2^^,^^6^ The risk of mortality increased linearly over the entire dose range, specially, with a 10% increase in mortality for every 10 units of RBC transfused and a 50% increase after 50 units.^6^ Therefore, our model may increase the precision of decision–making by taking into account the different volume of RBC transfusion, rather than simply predicting whether any transfusion will be required. In fact, our model may provide a more objective basis for stratifying patients by risk of transfusion, given that the STS transfusion risk factors^19^ cannot currently be weighted for individual patients.^20^ Our prediction model enables medical staff to stratify patients by their risk level, thus allowing them to optimize the allocation of blood resources and implement proactive strategies. The model also provides an early warning system to medical staff. For patients at risk of minor transfusion, routine perioperative monitoring and tests may be sufficient. However, for patients at a high risk of moderate to major transfusion, it is crucial to conduct timely preintervention patient assessments and optimize their hemoglobin and iron levels. Intravenous iron and other hematinics can be considered for treating anemia and iron deficiency.^4^ Additionally, various clinical measures have been implemented to reduce blood loss, such as conducting surgical hemostasis workshops, using hemostatic agents, employing blood-preserving and autologous blood salvage techniques, and minimizing the volumes of laboratory blood samples that are taken.^4^

In contrast to previous prediction models that were not validated externally or that did not perform well in such validation,^14^^,^^15^^,^^16^ our model has validated in another medical center that differed significantly from the development cohort. Notably, less accurate was observed in externally validated process, thus, our model requires further prospective validation and optimizing. Our validation cohort was significantly older; contained a greater proportion of women; showed higher rates of hyperlipemia, prior cardiac surgery, coronary artery disease, and aortic surgery; and showed lower preoperative hemoglobin levels. All these characteristics increase the risk of intraoperative transfusion^14^^,^^15^ and therefore help explain the much higher transfusion rate in the validation cohort. Despite these differences, our model predicted the three categories of transfusion volume well, suggesting that it is reliable and robust to different clinical contexts.

The predictive performance of all models may decay over time when transfusion strategies and surgical techniques change.^21^ For example, historically, the standard for the trigger of RBC transfusion trigger was “liberal” (hemoglobin level less than 10 g/dL or hematocrit below 30%).^22^ While, over the past years however, this arbitrary RBC transfusion trigger has gradually been changed and lowered toward a more “restrictive” one (the trigger of hemoglobin level was between 7 and 8 g/dL).^23^^,^^24^ Furthermore, blood salvage was relatively underused and blood salvage technology was only just being introduced into most of hospitals since 2009.^25^ While, at present, blood salvage has been a well–established technology of recovering shed blood during cardiac surgery, especially in on-pump procedures,^25^^,^^26^^,^^27^ and is widely accepted. Last but not the least, clinical guidelines began recommending retrograde autologous priming of the CPB circuit with a minimal volume.^27^ The aforementioned changes have a significant impact on blood transfusion volume and may substantially impact the robustness of the model. However, our model may be more robust than other models to such changes in practice, given that the 7 predictors in the model do not strongly depend on clinical management strategies. For the same reason, our model may be robust to differences in clinical practices across medical centers. Nevertheless, our model should be prospectively optimized and periodically updated to reflect current practices.

In conclusion, we have established and externally validated a model to predict minor, moderate, or major RBC transfusion in patients underwent on-pump cardiac procedures, based entirely on preoperative data easily extractable from electronic medical records. After further prospective improved and validation, our model might help support the implementation of transfusion-reducing interventions in high-risk individuals and has the potential to help improve patient safety and reduce healthcare costs. Notably, the development of our model is an important step toward personalized surgical blood orders.

### Limitations of the study

Certain intraoperative variables, such as CPB duration, CPB priming volume, and ultrafiltration strategy, may have confounded our analysis. We did not take these factors into account because we wanted to develop a model based only on preoperative data to ensure ease of use. Thus, the model could help clinicians stratify patients by transfusion risk and rationalized treatment decisions, preoperatively. For example, the assessment of preoperative anemia and determination of its etiology, use of synthetic antifibrinolytic agents (i.g., tranexamic acid) to reduce blood loss and transfusion during cardiac procedures, or routine use of RBC salvage in patients expected to have a high risk of major transfusion before surgery. At the same time, the transfusion-related risk for postoperative complications should be noted, especially for those fragile individuals. Second, we did not benchmark our new model against previously published ones. Third, the performance of the prediction model validated in the external validation cohort in this study, although modest, and the calibration of the model is limited. This limitation is likely due to the differing nature of the primary outcome, which is the units of transfusion required during on-pump cardiac surgery. This outcome may be influenced by the patient's functional condition, medical expertise, and geographic location. This raises caution regarding the generalizability of the prediction model. Fourth, all analyses were conducted after the data had been acquired; thus, the possibility of bias should be considered as it may have a significant impact on the results of current study. For instance, we excluded patients who died in the operating room as well as those who received an intra-aortic balloon pump or extracorporeal membrane oxygenation, both of which are cases that could potentially benefit from preoperative risk assessment. Therefore, the generalizability of the study may be limited. Lastly, rheumatic heart disease is the cause of up to 55% of patients with valvular heart disease in China.^28^ Therefore, caution should be exercised when the generalization of our findings in other regions with lower rheumatic heart disease prevalence, as well as in other types of cardiac surgery needs cautions. These highlight the need to optimize the model further, especially given the complicated nature of on-pump cardiac surgery and substantial differences in practices across medical centers.

## STAR★Methods

### Key resources table

**Table:** 

REAGENT or RESOURCE	SOURCE	IDENTIFIER
**Deposited data**		

**Clinical raw data**	This paper	https://doi.org/10.5281/zenodo.8125030

**Software and algorithms**		

R Software, 4.0.0	The R Foundation for Statistical Computing	https://www.r-project.org/
car (R-package)	Open source	https://r-forge.r-project.org/projects/car/
dplyr (R-package)	Open source	https://dplyr.tidyverse.org/
stringr (R-package)	Open source	https://stringr.tidyverse.org/
tableone (R-package)	Open source	https://github.com/kaz-yos/tableone
MASS (R-package)	Open source	https://www.stats.ox.ac.uk/pub/MASS4/
ROCR (R-package)	Open source	http://ipa-tys.github.io/ROCR/
pROC (R-package)	Open source	http://expasy.org/tools/pROC/
glmnet (R-package)	Open source	https://glmnet.stanford.edu/
ggplot2 (R-package)	Open source	https://ggplot2.tidyverse.org/
forester (R-package)	Open source	https://github.com/rdboyes/forester
nomogramEx (R-package)	Open source	https://cran.r-project.org/web/packages/nomogramEx/index.html
nomogramFormula (R-package)	Open source	https://github.com/yikeshu0611/nomogramFormula
chest (R-package)	Open source	https://cran.r-project.org/web/packages/chest/index.html
twang (R-package)	Open source	https://cran.r-project.org/web/packages/twang/index.html
nortest (R-package)	Open source	https://cran.r-project.org/web/packages/nortest/index.html
mice (R-package)	Open source	https://github.com/amices/mice
segmented (R-package)	Open source	https://cran.r-project.org/web/packages/segmented/index.html
questionr (R-package)	Open source	https://juba.github.io/questionr/
rms (R-package)	Open source	https://hbiostat.org/R/rms/
brant (R-package)	Open source	https://benjaminschlegel.ch/r/brant/
Codes in this paper	This paper	https://doi.org/10.5281/zenodo.8125003

### Resource availability

#### Lead contact

Further information and any related requests should be directed to and will be fulfilled by the lead contact, Qian Li (hxliqian@wchscu.cn).

#### Materials availability

This study did not generate new unique reagents.

### Experimental model and study participant details

The study was approved by the West China Hospital of Sichuan University (number 256/2017) and the Second Affiliated Hospital of Zhejiang University (number 096/2017). No protected health information was contained in database, a waiver of the requirement for informed consent was approved in the institutional review board approval. This study was registered at ClinicalTrials.gov (NCT04476134) and conducted in conformity with the principles outlined in the Declaration of Helsinki. There are no restrictions on ethnicity and gender in current study. This work was a secondary analysis of data from a retrospective observational cohort study.^29^

Development dataset comprises electronic health record data from 6820 consecutive patients at West China Hospital of Sichuan University in January 2011 through June 2017. We included patients who were at least 18 years old scheduled for valve re-placement or (or both) coronary artery bypass grafting (CABG) with cardiopulmonary bypass (CPB). Patients were excluded if combination surgery involving ascending aortic replacement, or they underwent emergency surgery (defined as surgery that is required to deal with an acute threat to life, within an interval of 24 hours), or died in the operating room. We additionally excluded cases that received an intra–aortic balloon pump or extracorporeal membrane oxygenation was applied in order to wean off pump during surgery.

The externally validated dataset comprises 1419 patients who met the abovementioned criteria at the Second Affiliated Hospital of Zhejiang University from September 2013 to June 2017.

### Method details

#### Study design

Current study followed the “Transparent reporting of a multivariable prediction model for individual prognosis or diagnosis” (TRIPOD) checklist,^30^ a copied version is provided in Supplemental of the Online Supplement. We studied elective cardiac surgical procedures with cardiopulmonary bypass (CPB) performed on adult patients at West China Hospital of Sichuan University (Chengdu, China) or the patients received surgical at the Second Affiliated Hospital of Zhejiang University (Zhejiang, China). The analysis plan of current study was written after the data had been accessed.

#### Perioperative management during cardiopulmonary bypass

The similar protocol of CPB was applied in two hospitals. Briefly, using cold 4:1 blood cardioplegia to achieve heart arrest. CPB was primed with 1500 mL liquid (including 4% succinylated gelatin solution of 1000 mL and 500 mL crystalline). Next, we set the blood flow ranged from 2.0 to 2.4 L/m^2^·min-1 in order to maintain mean arterial pressure at 50–80 mmHg during CPB. Besides, the nasopharyngeal temperature was maintained at 32–34 degree Celsius. 375 U/kg of heparin was applied in all patients to achieve systemic anticoagulation. Additional heparin could be used intermittently to keep activated clotting time longer than 480 seconds. Lastly, using protamine to neutralize heparin according to the initial heparin dose (in a 1:1 ratio) while wean from CPB.

The practices of transfusion were similar in the two hospitals: During CPB period, the trigger of RBC transfused was hemoglobin lower than 7 g/dL, or if the clinicians ordered transfusion according to the patient’s function condition. Other blood products could be administered if patients have bleeding events (i.e., ongoing bleeding or documented abnormal coagulation). Blood salvaged by suction from pericardial blood was returned to the CPB circuit during CPB procedures. Besides, using a bag containing sodium citrate to collect residual blood and neutralized by protamine, as well as transfused into the patient after weaning from CPB. Cell saver was routinely used in all cases.

#### Variables and outcomes measurement

Investigators using a standardized data collection form extracted data from the institutional cardiac surgery databases, individually. Data were collected on both patient– and surgery–specific variables, as well as preoperative medications, and laboratory findings (as shown in Table 1; Table S1).

We studies the association between the different volume of RBC transfusion during surgery and in–hospital adverse events, a composite of these three outcomes, new–onset stroke,^31^ myocardial infarction,^31^ or acute kidney injury.^32^ Briefly, new–onset stroke was diagnosed according to clinical report of brain imaging (computed tomography or magnetic resonance imaging), in association with new onset focal or generalized neurological deficit (defined as deficit in motor, sensory or coordination functions).^29^ Myocardial infarction was diagnosed according to electrocardiogram report of the occurrence of new Q waves, or ischemic ST changes in combination with abnormal postoperative troponin T levels (troponin T level > 0.5 mcg/L for coronary artery bypass grafting surgery, troponin T level > 0.8 mcg/L for valve surgery, and troponin T level > 1.0 mcg/L for combination CABG and valve procedures).^29^ Acute kidney injury was defined as creatinine rises 0.3 mg/dl or greater within 48 hours or greater than 1.5 times baseline within first 7-day^31^ Patients who experienced two or three of these events were classified under the event that occurred first.

The outcome variable in the model was the different volume of RBC transfusion during surgery. Minor transfusion was defined as up to 2 units of RBCs, 1 unit of RBCs equals 200 mL; moderate transfusion, as greater than 2 units and up to 4 units; and major transfusion, as more than 4 units. Major transfusion corresponded to a units of approximately 1000 mL, which has been shown its inked to significantly increased risk of short- and long-term morbidity and mortality.^33^^,^^34^

### Quantification and statistical analysis

#### Prediction model development and external validation

Using the proportional ordinal logistic model to construct the prediction model. Two algorithms were used to select candidate predictors during the model development process. In one approach, all candidate predictors with *P* < 0.10 in the univariable analysis were included multivariable logistic regression. In the second approach, we applied the least absolute shrinkage or selection operator (LASSO) to select potential predictors and validated by 10-fold cross-validation to avoid overfitting. Using the Nagelkerke R^2^ (for which larger values are better), Bayesian information criterion and root mean square error (both values were smaller, better) to determining the performance of these methods.^35^^,^^36^ Optimal predictor selection algorithm was determined by Occam’s Law of Razor, which applied the minimum predictors to reach the best performance.

Restricted cubic spline (RCS) models with 3–5 knots were used to transform confirmed nonlinear continuous predictors into categorical predictors,^37^ which were optimally fitted according to the likelihood ratio test and Bayesian information criterion. Break-point analysis for optimum cut-off values was also employed using the method proposed by previously published method. The collinearity between predictors was determined according to the variance inflation factor (VIF). The discriminative ability of the prediction model was assessed in three ways, based on 1) areas under the receiver operating characteristic curves (AUCs),^38^ 2) a calibration curve was computed to assess graphically the calibration of model-predicted probabilities, and 3) then, prediction model performance was also evaluated in terms of the calibration slope (the smaller values suggesting poor calibration, ideal value is 1) and Brier score (the values more than 0.2 suggesting poor calibration, and ideal is 0).^35^^,^^36^

We computed a decision curve analysis to validate the prediction model’s clinical usefulness, this method can evaluate and compare the actual and model-prediction probability by integrating the clinical consequences of false positive and negative results, which quantifying the net benefits at different threshold probabilities.^39^

Then, the best–performing model was validated on the external validation cohort. We basically applied the same methods to assess the model performance.

#### The association of transfusion volume and postoperative composite adverse events

We assessed the relationship of different RBC transfusion volume during surgery and postoperative composite adverse events in development cohort. The gradient boosted model (GBM)–based inverse probability of treatment weighting (IPTW) method was employed for adjusting confounders.^40^ GBM-IPTW controls for confounding by eliminating imbalances in measured covariates between the study groups. The absolute standardized mean difference was calculated to evaluate the balancing effectiveness of the IPTW; values below 0.20 were considered satisfactory.^40^ For confounder selection in IPTW algorithm, baseline unbalanced variables were considered as the measured confounding covariates.

The potential dose–dependence of the relationship between different transfusion volume and composite adverse events was tested by treating median units for minor, moderate, or major transfusion subgroups as a continuous variable in the logistic regression model.

#### Statistical analysis

All the analyses were performed using R software, version 4.0.0 (The R Foundation for Statistical Computing, https://www.r-project.org/). Statistical significance is considered when the 2-sided *P*-value is less than 0.05.

Continuous variables were presented mean (SD) or median with interquartile ranges (25th, 75th percentile), as appropriate. Categorical variables were shown as count of number with percentage (%). Comparison between subgroups were performed using Student’s *t*, Welch’s *t*, Wilcoxon rank–sum (for continuous variables), and chi-square or Fisher exact test (for categorical variables). Those data with missing rate of greater than 10% were excluded in our study. For those with less than 10% rate, multiple imputation by chained equation algorithm based on MICE package (https://doi.org/10.18637/jss.v045.i03) was applied to resolve them. Missing proportion and pattern were also visualized (Figure S2).

## Data Availability

• The data generated in this study is available on Zenodo. DOIs are listed in the key resources table. Currently, the data of our study are not available to public for data protection and privacy policy. But we welcome clinicians and researchers contact us, for initiating collaborative projects or joining us as a new recruitment center. Please contact corresponding authors Lei Du (dulei@scu.edu.cn) or Qian Li (liqian@wchscu.cn) for collaboration requests. • The version of source code used for the preparation of the manuscript is available on Zenodo. DOIs are listed in the key resources table. • Any additional information required to reanalyse the data reported in this paper is available from the lead contact upon request.

## Supplemental information

Document S1. Figures S1–S9, Tables S1, S2, and S4, and Data S1

Table S3. Uni- and multivariable results based on the ordinal logistic model for identifying predictors of the units of transfusion in the development cohort, related to Table 2, Figures 1 and 2
