# Supplementary material for: A model based on electronic health records to predict transfusion events in on-pump cardiac surgery
Source: iScience. 2023 Sep 1;26(10):107798. doi: 10.1016/j.isci.2023.107798 (PMC10514444; doi:10.1016/j.isci.2023.107798)
Supplement: Document S1. Figures S1–S9, Tables S1, S2, and S4, and Data S1 [file mmc1.pdf]

iScience, Volume *26*

Supplemental information

A model based on electronic health records to predict transfusion events in on-pump cardiac surgery

Dong Xu Chen, Yi Shun Wang, Min Yan, Lei Du, and Qian Li

Data S1/Methods S1: Completed Transparent Reporting of a Multivariable Prediction Model for Individual Prognosis (TRIPOD) checklist, related to related to Table 1, Summary, Results, and STAR Methods.

Section/Topic	Item	Checklist item	Completed?
Title	1	Identify the study as developing and/or validating a multivariable prediction model, the target population, and the outcome to be predicted.	Yes
Abstract	2	Provide a summary of objectives, study design, setting, participants, sample size, predictors, outcome, statistical analysis, results, and conclusions.	Yes
Background	3a	Explain the medical context (including whether diagnostic or prognostic) and rationale for developing or validating the multivariable prediction model, including references to existing models.	Yes
Objectives	3b	Specify the objectives, including whether the study describes the development or validation of the model, or both.	Yes
Source of Data	4a	Describe the study design or source of data (e.g., randomized trial, cohort, or registry data), separately for the development and validation data sets, if applicable.	Yes
	4b	Specify the key study dates, including start of accrual; end of accrual; and, if applicable, end of follow-up.	Yes
Participants	5a	Specify key elements of the study setting (e.g., primary care, secondary care, general population) including number and location of centers.	Yes
	5b	Describe eligibility criteria for participants.	Yes
	5c	Give details of treatments received, if relevant.	N/A because retrospective study.
Outcome	6a	Clearly define the outcome that is predicted by the prediction model, including how and when assessed.	Yes
	6b	Report any actions to blind assessment of the outcome to be predicted.	N/A because retrospective study.
Predictors	7a	Clearly define all predictors used in developing the multivariable prediction model, including how and when they were measured.	Yes
	7b	Report any actions to blind assessment of predictors for the outcome and other predictors.	Yes
Sample Size	8	Explain how the study size was arrived at.	Yes
Missing Data	9	Describe how missing data were handled (e.g., complete-case analysis, single imputation, multiple imputation) with details of any imputation method.	Yes
Statistical Analysis Methods	10a	Describe how predictors were handled in the analyses.	Yes
	10b	Specify type of model, all model-building procedures (including any predictor selection), and method for internal validation.	Yes
	10c	For validation, describe how the predictions were calculated.	Yes

	10d	Specify all measures used to assess model performance and, if relevant, to compare multiple models.	Yes
	10e	Describe any model updating (e.g., recalibration) arising from the validation, if done.	N/A because no model updating.
Risk Groups	11	Provide details on how risk groups were created, if done.	N/A, not done.
Development vs. validation	12	For validation, identify any differences from the development data in setting, eligibility criteria, outcome, and predictors.	Yes
Participants	13a	Describe the flow of participants through the study, including the number of participants with and without the outcome and, if applicable, a summary of the follow-up time. A diagram may be helpful.	Yes
	13b	Describe the characteristics of the participants (basic demographics, clinical features, available predictors), including the number of participants with missing data for predictors and outcome.	Yes
	13c	For validation, show a comparison with the development data of the distribution of important variables (demographics, predictors, and outcome).	Yes
Model Development	14a	Specify the number of participants and outcome events in each analysis.	Yes
	14b	If done, report the unadjusted association between each candidate predictor and outcome.	Yes
Model Specification	15a	Present the full prediction model to allow predictions for individuals (i.e., all regression coefficients, and model intercept or baseline survival at a given time point).	Yes
	15b	Explain how to use the prediction model.	Yes
Model Performance	16	Report performance measures (with CIs) for the prediction model.	Yes
Model Updating	17	If done, report the results from any model updating (i.e., model specification, model performance).	Yes
Limitations	18	Discuss any limitations of the study (such as nonrepresentative sample, few events per predictor, missing data).	Yes
Interpretation	19a	For validation, discuss the results with reference to performance in the development data, and any other validation data.	Yes
	19b	Give an overall interpretation of the results, considering objectives, limitations, results from similar studies, and other relevant evidence.	Yes
Implications	20	Discuss the potential clinical use of the model and implications for future research.	Yes
Supplementary Information	21	Provide information about the availability of supplementary resources, such as study protocol, Web calculator, and data sets.	Yes
Funding	22	Give the source of funding and the role of the funders for the present study.	Yes

Supplemental table S1. Additional baseline characteristics of patients in the development and external validation cohorts, related to Table 1.

Characteristics	Development cohort N=6820	External validation cohort N=1419	p-value
**Operative indication, n (%)**			
Simple valvular disease	1303 (19.1)	373 (26.3)	<0.001
Complex valvular disease	4802 (70.4)	642 (45.2)	<0.001
CABG	554 (8.1)	236 (16.6)	<0.001
Combined CAD and valvular disease	108 (1.6)	128 (9.0)	<0.001
Infective endocarditis	48 (0.7)	27 (1.9)	<0.001
**Medications, n (%)**			
β–adrenergic receptor blocker	848 (12.4)	212 (14.9)	0.011
Clopidogrel	30 (4.4)	164 (11.6)	<0.001
Aspirin	35 (0.5)	257 (18.1)	<0.001
Inotropic	2211 (32.4)	129 (9.1)	<0.001
Anticoagulants	966 (14.2)	357 (25.2)	0.011
Diuretics	5487 (80.5)	240 (16.9)	<0.001
**Preoperative laboratory findings**			
LVEF (%), median [IQR]	62.00 [56.00, 67.00]	61.00 [55.00, 67.00]	<0.001
Hemoglobin (g/dL), mean (SD)	13.49 (1.76)	13.01 (1.87)	<0.001
RBC count (x 10^12/L), mean (SD)	4.56 (0.59)	4.34 (0.67)	<0.001
WBC count (x 10^9/L), mean (SD)	6.08 (1.77)	6.32 (2.24)	<0.001
PLT count (x 10^9/L), mean (SD)	156.49 (60.61)	178.49 (66.88)	<0.001
PT (s), median [IQR]	11.90 [11.20, 12.70]	13.40 [12.90, 14.40]	<0.001
APTT (s), median [IQR]	28.70 [26.10, 31.70]	38.20 [35.20, 42.20]	<0.001
Fibrinogen (g/L), median [IQR]	2.74 [2.34, 3.24]	3.17 [2.68, 3.75]	<0.001
INR, median [IQR]	1.04 [0.99, 1.11]	1.04 [0.98, 1.13]	0.802
Albumin (g/L), mean (SD)	42.49 (4.02)	39.57 (4.23)	<0.001
ALT (IU/L, median [IQR]	22.00 [16.00, 32.00]	20.00 [14.00, 29.50]	<0.001
Total bilirubin (μmol/L), median [IQR]	42.49 (4.02)	39.57 (4.23)	<0.001
BUN (mmol/L), median [IQR]	5.65 [4.60, 6.90]	5.85 [4.78, 7.18]	<0.001
Scr (umol/L), median [IQR]	72.00 [62.58, 83.30]	66.00 [56.00, 79.00]	<0.001

Cys–C (mg/L), median [IQR]	1.03 [0.92, 1.16]	1.14 [0.99, 1.36]	<0.001
Blood glucose (mmol/L), mean (SD)	5.21 (1.19)	5.33 (1.46)	0.003

Continuous variables were reported as mean (standard deviation) or median (interquartile range); categorical variables, as n (%).

[*] Patients at West China Hospital of Sichuan University.

[#] Patients at the Second Affiated Hospital of Zhejiang University.

ALT, alanine aminotransferase; APTT, activated partial thromboplastin time; BUN, blood urea nitrogen; CAD, coronary artery disease; CABG, coronary artery bypass grafting; Cys–C, Cystatin C; INR, international normalized ratio; IQR, interquartile range; LVEF, left ventricular ejection fraction; PLT, platelet; PT, prothrombin time; RBC, red blood cell; SD, standard deviation; Scr, serum creatinine; WBC, white blood cell.

Supplemental Table S2. Comparison of outcomes between the development and external validation cohorts, related to Table 1.

Outcome, n (%)	Development cohort					External validation cohort					p-value
	Total N=6820	No transfusion N=5601	Minor transfusion N=809	Moderate transfusion N=341	Major transfusion N=69	Total N=1419	No transfusion N=670	Minor transfusion N=253	Moderate transfusion N=266	Major transfusion N=230	
**Composite outcome**	1003 (14.7)	761 (13.6)	141 (17.4)	80 (23.5)	21 (30.4)	630 (44.4)	221 (33.0)	116 (45.8)	140 (52.6)	153 (66.5)	<0.001
AKI	988 (14.5)	751 (13.4)	139 (17.2)	77 (22.6)	21 (30.4)	624 (44.0)	219 (32.7)	114 (45.1)	138 (51.9)	153 (66.5)	<0.001
Stroke	19 (0.3)	13 (0.3)	1 (0.1)	4 (1.2)	1 (1.4)	19 (1.4)	2 (0.3)	2 (0.8)	2 (0.8)	7 (3.0)	0.002
Myocardial infarction	11 (0.2)	9 (0.2)	1 (0.1)	1 (0.3)	0 (0.0)	1 (0.1)	0 (0.0)	1 (0.4)	0 (0.0)	0 (0.0)	0.665

Values are n (%), unless otherwise noted.
AKI, acute kidney injury.

Supplemental Table S4. Parallel assumption in ordinal logistic regression, related to Table 2, Figure 1, and Figure 2.

**Variables**	χ^2	**df**	**probability**
Omnibus	38.32	26	0.057
Age (years)			
30~50	1.92	2	0.382
50~70	1.39	2	0.500
>70	1.29	2	0.525
Male	3.74	2	0.154
BMI (kg/m^2)			
18.5~23.9	0.02	2	0.991
23.9~27.9	0.16	2	0.924
>27.9	0.10	2	0.950
Preoperative hemoglobin (g/dL)			
8~11	0.21	2	0.900
11~13	0.53	2	0.769
>13	0.68	2	0.711
History of atrial fibrillation	0.71	2	0.303
Prior cardiac surgery	1.65	2	0.700
**Operative indications**			
Coronary artery bypass grafting	2.39	2	0.303

Supplemental Figure. S1. Flow chart of study design, related to STAR Methods.

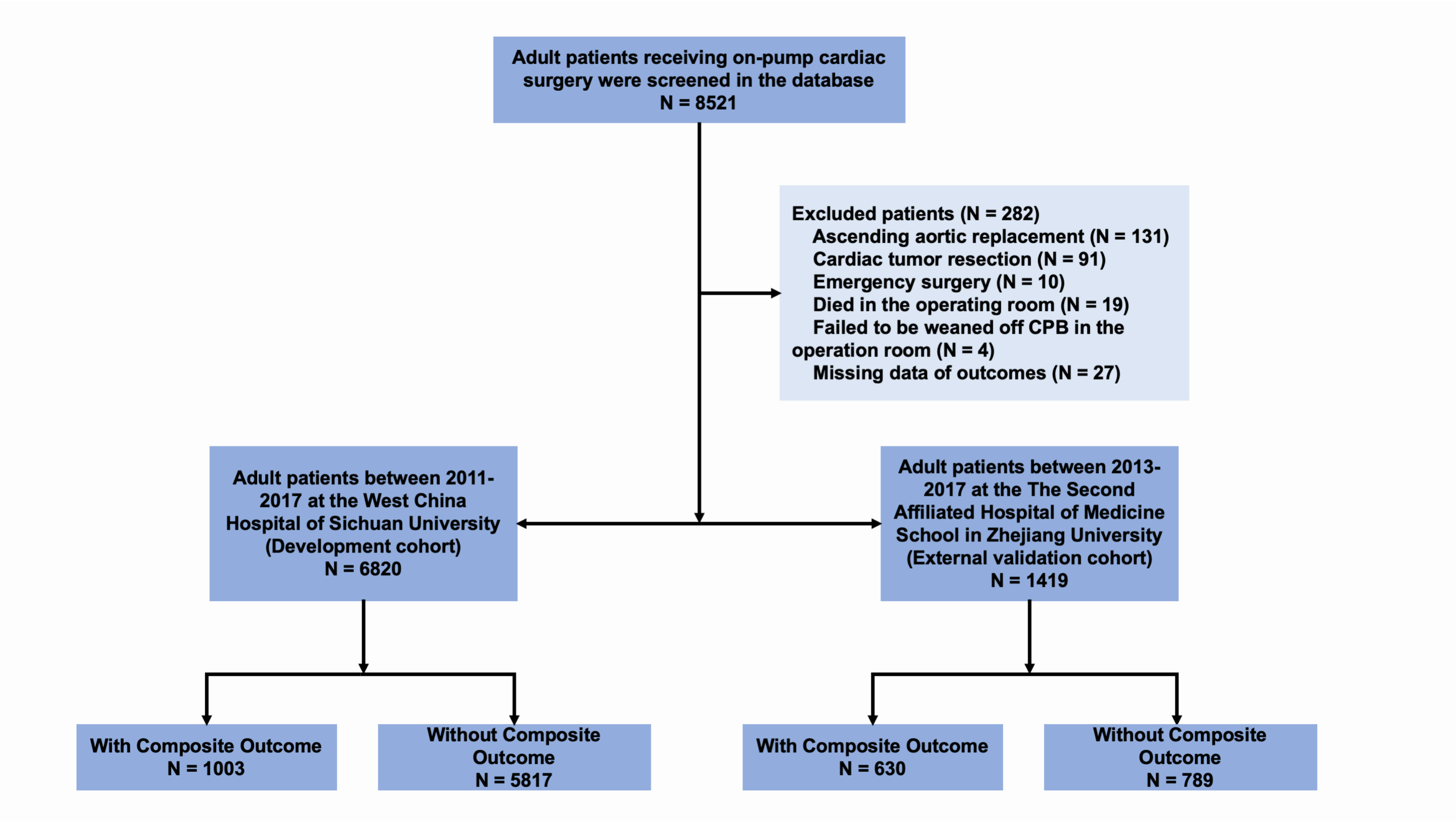

Supplemental Figure S2. Visual representation of missing data for (A–B) categorical variables in the (A) development cohort and (B) external validation cohort, or missing data for (C–D) continuous variables in the (C) development cohort and (D) external validation cohort.
The histograms show the proportions of missing data for each variable. In the pattern plots, variables with complete data are shown in blue, while those with missing data are shown in grey, related to Table 1 and STAR Methods.

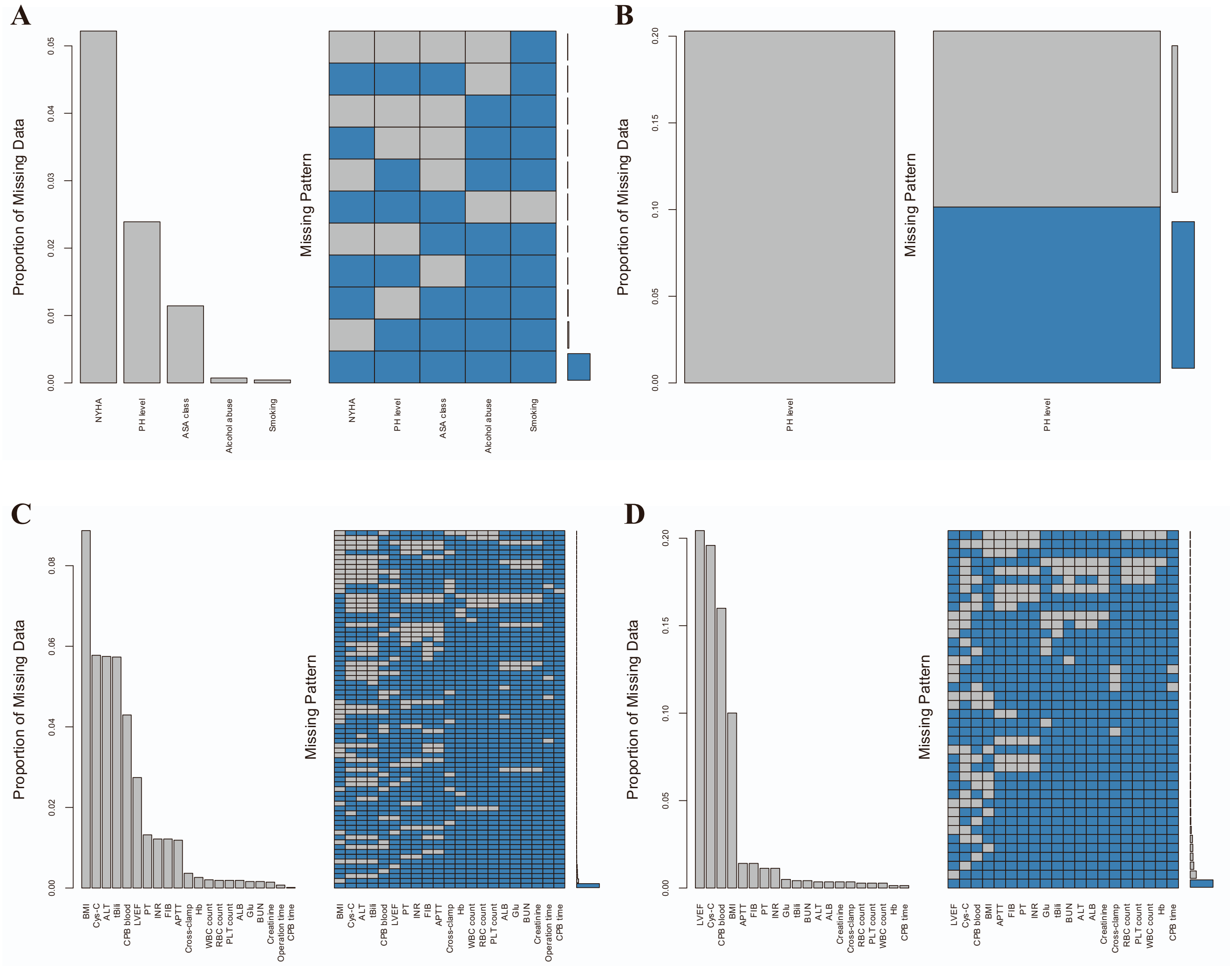

Supplemental Figure S3. Feature variable selection using least absolute shrinkage and selection operator (LASSO) regression in the development cohort, related to Table 2 and Figure 2.

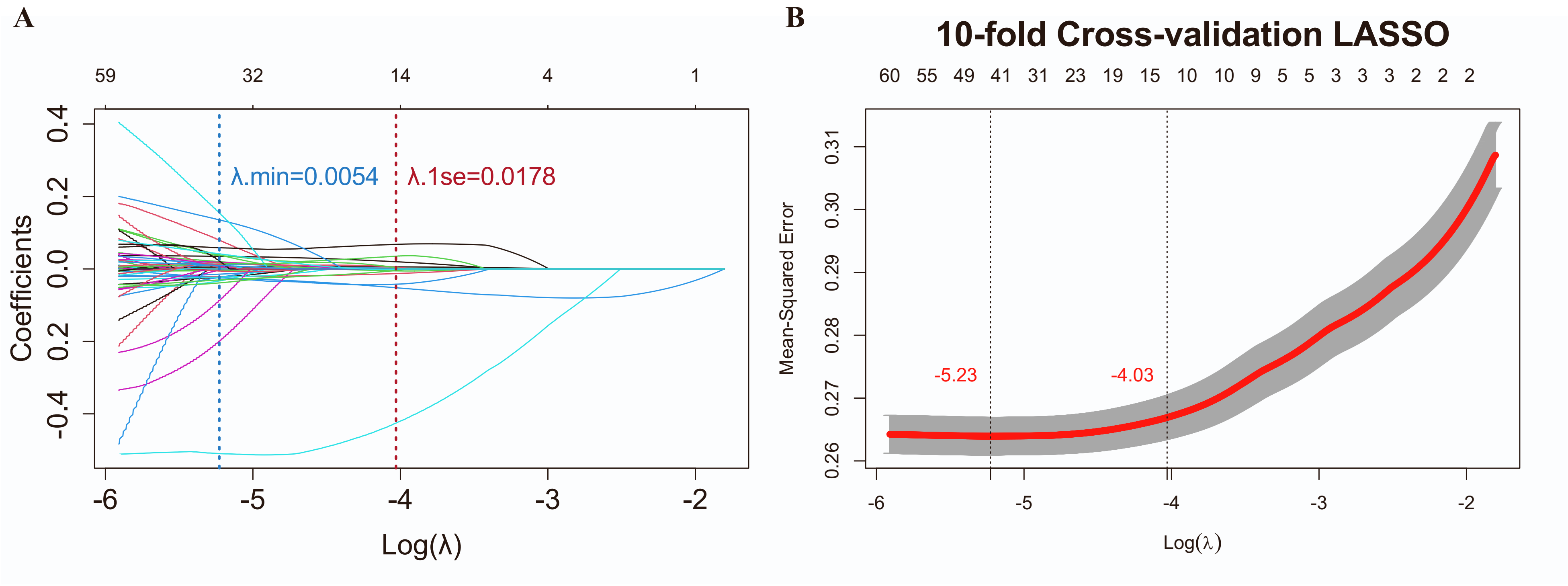

(A) A LASSO coefficient profile of all feature variables against the log (λ) sequence. The blue and red dotted vertical lines indicate, respectively, the log(λ_{min}) and log(λ_{1se}). The resulting 10 predictors with non-zero coefficients were identified based on the log (λ_{1se}) value.
(B) LASSO−based ordinal logistic model with 10-fold cross-validation based on the minimal mean squared error (MSE) was employed to find the optimal parameter (λ). The MSE *vs* log (λ) is shown in the plot. Two dotted vertical lines indicate the optimal λ values based, respectively, on the criterion of minimal MSE (λ_{min}) or the criterion of one standard error of the minimum (λ_{1se}). The log (λ_{1se}) of −3.49 and λ_{1se} of 0.019 were considered optimal.

Supplemental Figure S4. Forest plots for predictors of transfusion based on each of the two selection methods: (A) uni- and multivariate analysis with 7 predictors, (B) LASSO regression with 14 predictors, related to Table 2 and Figure 2.

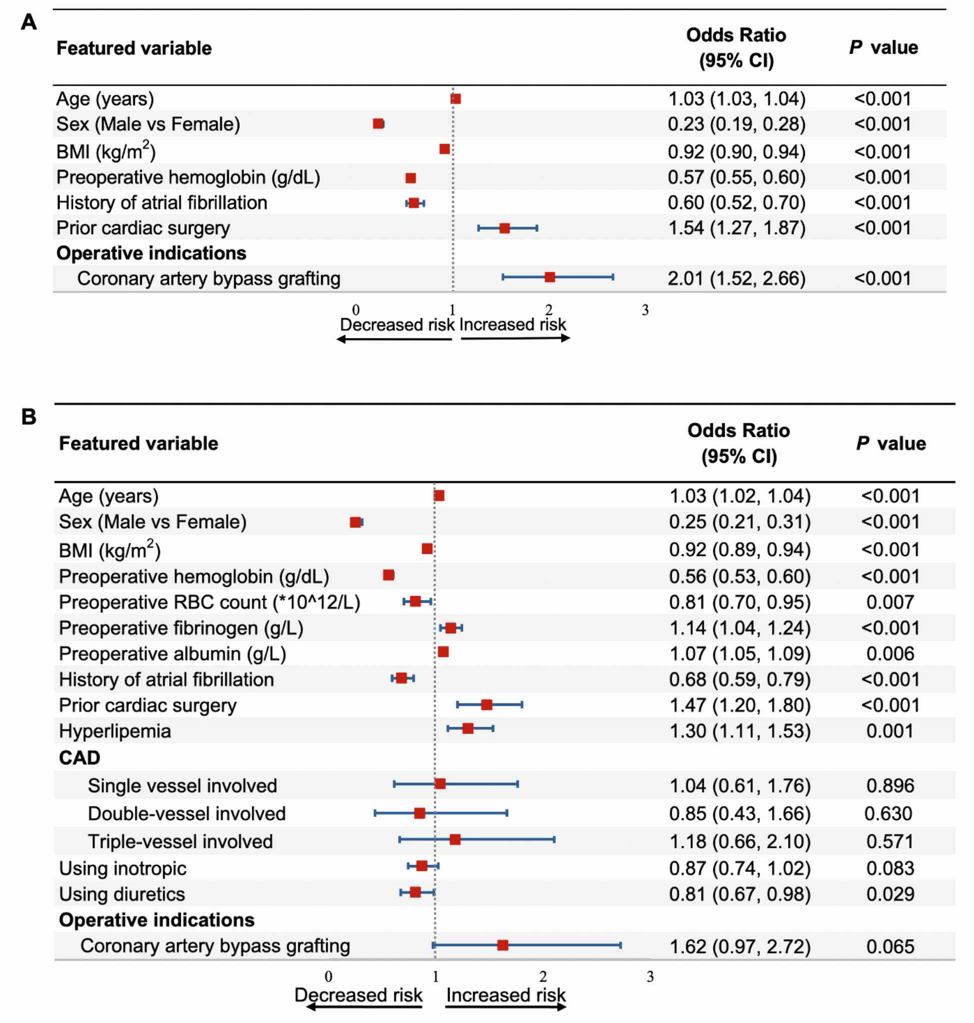

BMI, body mass index; CAD, coronary artery disease; CI, confidence interval; RBC, red blood cell.

Supplemental Figure S5. Non–linear associations of the volume of red blood cell transfusion with (A) age, (B) body mass index (BMI), or (C) preoperative hemoglobin (Hb) level in the development cohort, related to Table 2, Figure 1, and Figure 2.

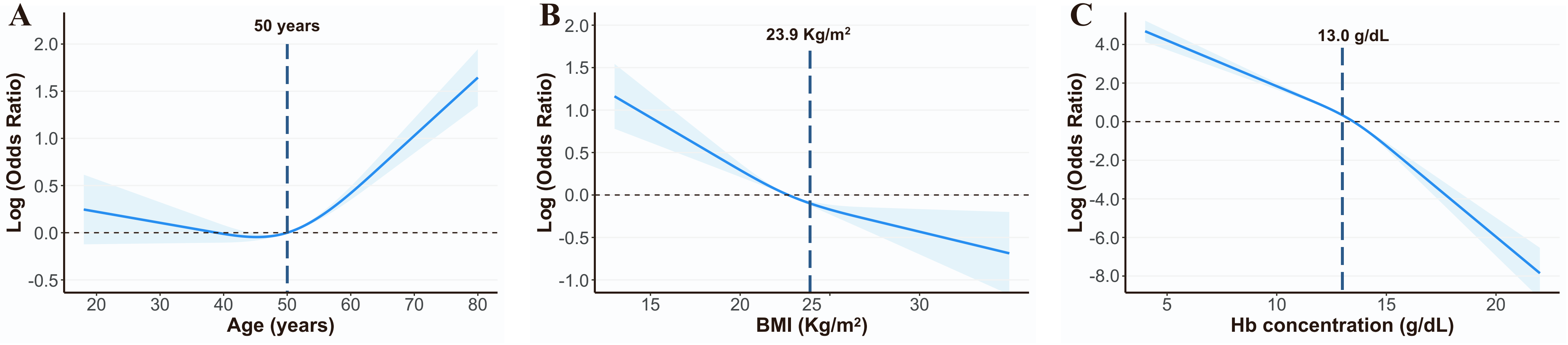

A univariate ordinal logistic model was used based on restricted cubic splines with 3–5 knots. The light blue shading depicts 95% confidence intervals. The median value of each variable is indicated with a red dashed line. For all three variables, sensitivity analyses identified 3–knot restricted cubic spline models as optimal, in terms of the likelihood ratio test and Bayesian information criterion.

BMI, body mass index; Hb, hemoglobin; RCS, restricted cubic spline.

Supplemental Figure S6. Calibration curves for testing the stability of the prediction model in the development and external validation cohorts, related to Figure 2.

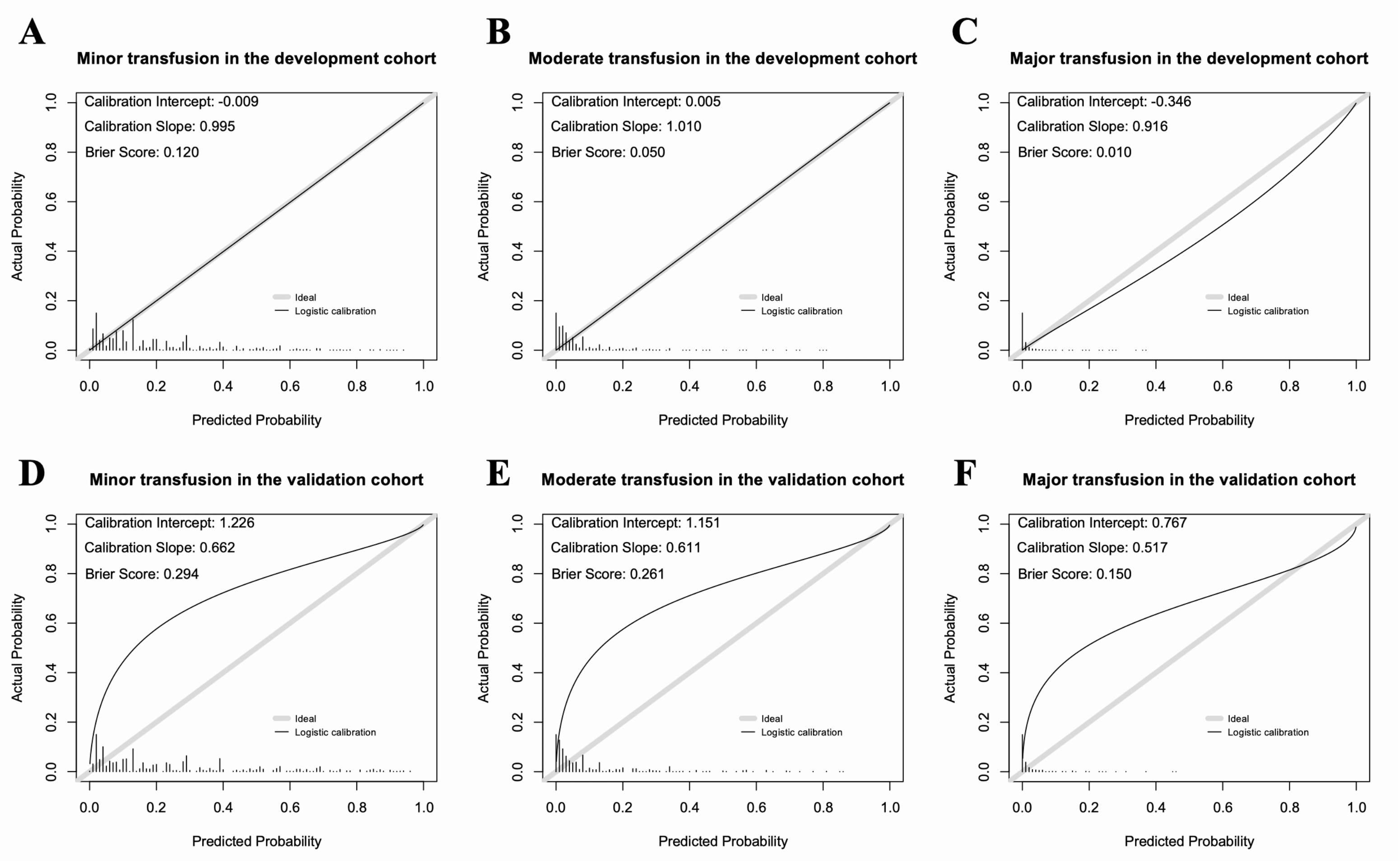

Logistic calibration curves are indicated in solid black lines. Ideal reference lines are depicted as gray dotted lines. Statistical results are shown at the upper left of each plot.

Supplemental Figure S7. Decision curve analysis of the prediction model for the development and external validation cohorts, related to Figure 2.

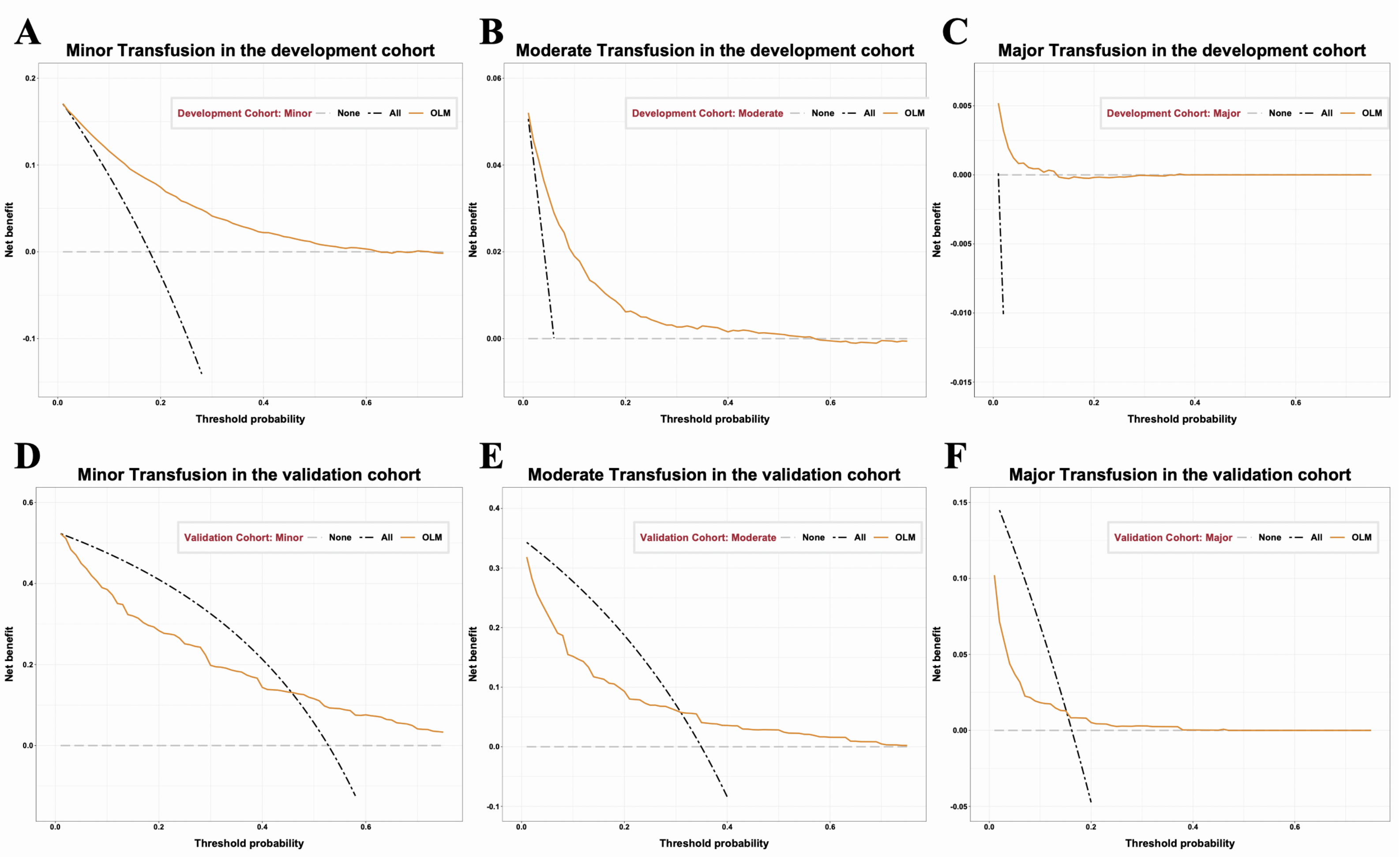

Net benefit of using a model to predict the volume of red blood cell transfusion as compared with strategies of 'treat all patients' or 'treat no patient' for different thresholds. Net benefits (orange solid lines) of the OLM-based model are shown as a function of the sequential probability thresholds for all three transfusion volumes.

Supplemental Figure S8. (A) Relative influence of each potential confounder, based on weights estimated by the GBM–IPTW method. Confounding covariates without any influence are not shown. (B) Balance diagnostic plot based on the GBM-IPTW method. (Related to STAR Methods).

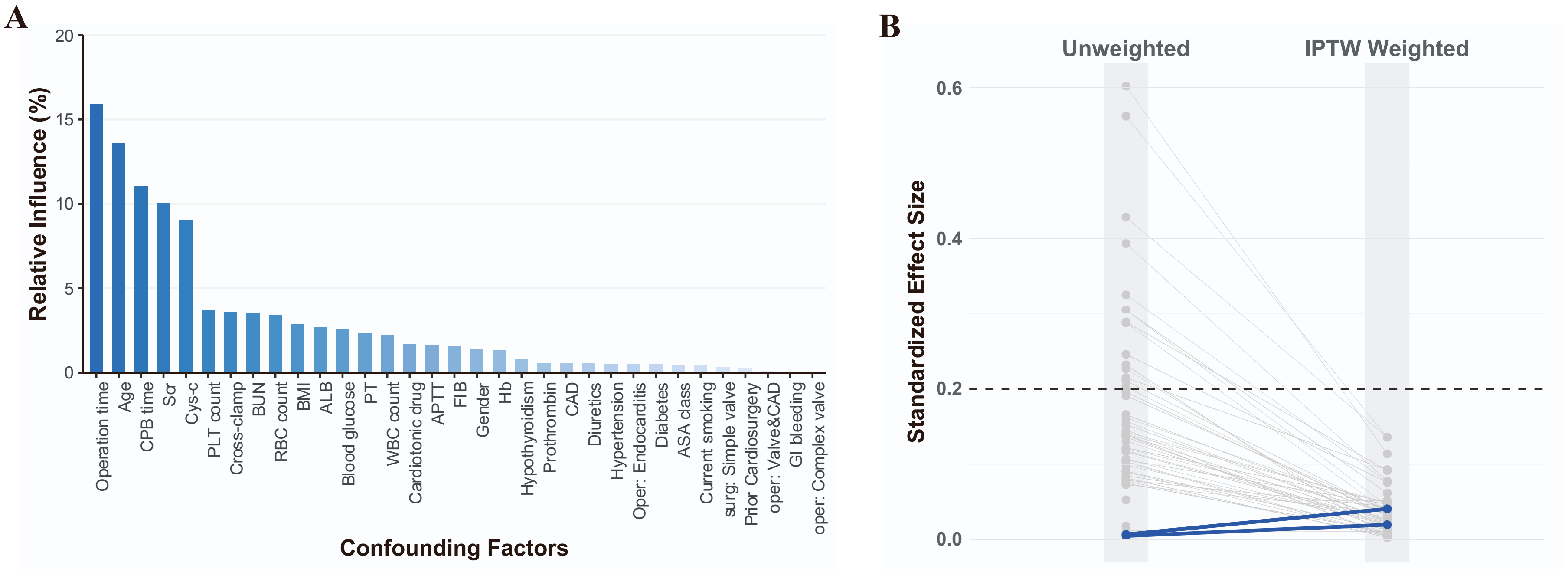

Blue lines indicate the increase in confounding due to IPTW weighting. The grey horizontal dash line indicates the cut-off in absolute standardized mean difference of 0.20. GBM, gradient boosted model; IPTW, inverse probability of treatment weighting.

Supplemental Figure S9. Associations between the volume of red blood cell transfusion and in-hospital composite adverse events, related to STAR Methods.

Variable	No. of patients with event/Total No. of patients	Unweighted		IPTW weighted	
		Odds Ratio (95% CI)	*P* value	Odds Ratio (95% CI)	*P* value
No transfusion	761/5601 (13.6)	Ref		Ref	
Minor transfusion	141/809 (17.4)	1.34 (1.11, 1.63)	0.003	1.33 (1.19, 1.48)	<0.001
Moderate transfusio	80/341 (23.5)	1.95 (1.49, 2.52)	<0.001	1.70 (1.46, 1.98)	<0.001
Major transfusion	21/69 (30.4)	2.78 (1.62, 4.61)	<0.001	1.92 (1.42, 2.63)	<0.001
		***P* for trend: <0.001**		***P* for trend: <0.001**	

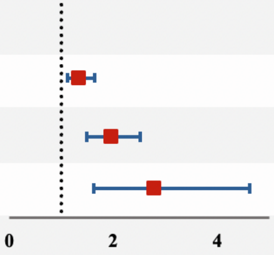

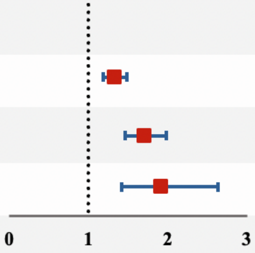

IPTW, inverse probability of treatment weight; CI, confidence interval.
